# Positioning, Navigation, and Book Accessing/Returning in an Autonomous Library Robot using Integrated Binocular Vision and QR Code Identification Systems

**DOI:** 10.3390/s19040783

**Published:** 2019-02-14

**Authors:** Xiaojun Yu, Zeming Fan, Hao Wan, Yuye He, Junye Du, Nan Li, Zhaohui Yuan, Gaoxi Xiao

**Affiliations:** 1 School of Automation, Northwestern Polytechnical University, Xi’an 710072, China; XJYU@nwpu.edu.cn (X.Y.); wanhaonwpu@mail.nwpu.edu.cn (H.W.); 15529301867@mail.nwpu.edu.cn (Y.H.); dujunye@mail.nwpu.edu.cn (J.D.); nan.li@nwpu.edu.cn (N.L.); yuanzhh@nwpu.edu.cn (Z.Y.); 2 School of Electrical and Electronic Engineering, Nanyang Technological University, 50 Nanyang Avenue, Singapore 639798, Singapore; EGXXIAO@ntu.edu.sg

**Keywords:** Robots, Positioning and navigation, QR code image, Imaging processing

## Abstract

With rapid advancements in artificial intelligence and mobile robots, some of the tedious yet simple jobs in modern libraries, like book accessing and returning (BAR) operations that had been fulfilled manually before, could be undertaken by robots. Due to the limited accuracies of the existing positioning and navigation (P&N) technologies and the operational errors accumulated within the robot P&N process, however, most of the current robots are not able to fulfill such high-precision operations. To address these practical issues, we propose, for the first time (to the best of our knowledge), to combine the binocular vision and Quick Response (QR) code identification techniques together to improve the robot P&N accuracies, and then construct an autonomous library robot for high-precision BAR operations. Specifically, the binocular vision system is used for dynamic digital map construction and autonomous P&N, as well as obstacle identification and avoiding functions, while the QR code identification technique is responsible for both robot operational error elimination and robotic arm BAR operation determination. Both simulations and experiments are conducted to verify the effectiveness of the proposed technique combination, as well as the constructed robot. Results show that such a technique combination is effective and robust, and could help to significantly improve the P&N and BAR operation accuracies, while reducing the BAR operation time. The implemented autonomous robot is fully-autonomous and cost-effective, and may find applications far beyond libraries with only sophisticated technologies employed.

## 1. Introduction

With the rapid advancement of artificial intelligence, robots are playing an increasingly important role in both industry and daily routines [1]. More and more robots are being developed and widely deployed to undertake various jobs that had been fulfilled manually before [2,3]. For example, in modern libraries, mobile robots are being employed to fulfill the tedious yet simple book accessing and returning (BAR) tasks [4,5]. To enable such high-precision functions, however, numerous technologies (e.g., sensing, actuation, manipulation, locomotion, environmental interaction, positioning and navigation (P&N), and human-robot interaction) are involved. Among all those required technologies, the indoor robot P&N technologies are the key factors in determining the accuracies of robot movements, book identification, and accessing and returning operations.

The current navigation technologies typically include satellite navigation, magnetic navigation, sensor navigation, inertial navigation, and visual navigation [6,7]. Satellite and magnetic navigation technologies are two conventional ways of navigation, yet are seldom utilized for indoor P&Ns. This is because the satellite signals can not penetrate through the buildings, while the magnetic navigation guide lines are expensive and difficult to expand or replace once located [8]. Sensor navigation, including infrared, laser, and ultrasound navigation, are typically based on nonvisual sensors to measure the transmitting and reflecting signals from the objects. Owing to their limited navigation accuracies, navigation distances, and high prices, sensor navigation is commonly adopted for military purposes [9,10]. Inertial navigation is a sophisticated way for autonomous navigation without relying on any external information. However, its cost is high and it also requires a large amount of time for calibration before use, due to the influences of the accumulated navigation errors [11]. Vision navigation, including monocular vision, binocular vision, and multi-vision, is based on camera systems to collect surrounding information to establish environment maps for P&Ns [12]. Due to the limited field of view (FOV) of the cameras, however, vision navigation suffers from a lack of accuracy in large distance positioning, and it is typically utilized for indoor navigation [13,14].

To address the limited accuracy issues with these existing navigation technologies, new schemes—simultaneously utilizing two or more existing navigation technologies—have been proposed in the literature [11,15,16,17,18,19]. Based on integrated inertial and binocular vision navigation technologies, Wang et al. proposed a global map generation method to tackle the difficulties in indoor navigation map constructions [16]. While by utilizing the simultaneous vision positioning and indoor map construction technologies to generate real-time incremental maps, authors in [17] showed that the accumulated navigation system errors could be reduced effectively. Recently, Wang et al. adopted a visual odometer [17,18] to achieve unmanned aerial vehicle (UAV) positioning based on the visual optical flow information and inertial sensing data [19]. However, it is worth noting that the amount of data to be processed with the integrated visual and inertial navigation technologies is huge, which thus limits the accuracy of the library digital maps generated for real-time applications.

To further improve the digital map accuracy while reducing the computational load, quick response (QR) code-based technology has also been introduced for mobile robot P&Ns [20,21,22,23]. QR codes are well-known for their low cost, large data storage, robustness against damages, and easy productivity/readability, etc., and they have been widely deployed in various cases in recent years. Eimon et al. pioneered the research by utilizing QR codes as personal identification tags and developed a human-tracking robot for services in public places like airports or supermarkets [20]. Later, Suriyon et al. proposed to utilize QR codes as landmarks for a visitor guide robot traversing between two specified locations [23], and Zhang et al. implemented an indoor mobile robot localization and navigation mechanism by utilizing QR codes to provide global location information and robot pose references [21]. However, it is worth noting that all these developed robots either have only fixed targets or could only traverse between fixed locations without route re-planning functions, even though obstacles may block the robot trajectory occasionally in indoor environment. Furthermore, due to the limited FOV of the QR code scanners, those robots may also suffer from certain practical issues, such as the rediscovery issue in case of object missing, the QR code running outside issue at turning points, QR code access issues caused by light/sheltering conditions, and so on. Although some of those issues can be addressed by faster QR code scanners [22], effective mechanisms are still needed for the ever-increasing high-precision mobile robot applications.

To benefit from the advantages of both binocular vision navigation and QR code identification technologies, we propose, for the first time (to the best of our knowledge), to integrate these two technologies together to improve the mobile robot P&N accuracies, and also construct an autonomous robot to implement the high-precision BAR operations, based on such a technology combination. In this paper, the binocular vision system is utilized to fulfill the rapid QR code region localization, dynamic digital map construction, autonomous robot P&N, as well as obstacle identification and avoiding functions. While the QR code identification technique is used to identify those QR code labels, and then access their contents for both robot operational error eliminations and robotic arm BAR operation calculations. With those two technologies working together, the robot is able to establish a high-precision library digital map, and then identify and avoid those obstacles to fulfil the real-time autonomous route-planning functions. Meanwhile, by accessing the QR code labels, it is also able to update the digital map frequently to eliminate the errors accumulated during its P&N process and, thus, realize the high-precision BAR operations. Both simulations and experiments have been conducted to verify the effectiveness of such a technology combination and the robot functionalities in different cases.

The remainder of this paper is organized as follows. Section 2 presents the robot structure design and its binocular vision system. Section 3 describes the proposed binocular vision and QR code identification technology integration based robot P&N and BAR operation mechanisms. Section 4 presents the experiments conducted for mechanism and system verifications, and Section 5 concludes the paper.

## 2. Library Robot Structure and Its Vision System

### 2.1. Robot Structure Design

**Figure 1:**
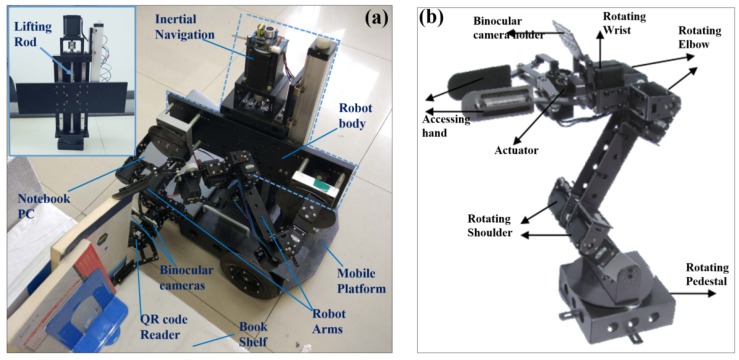
Structure design of the library mobile robot for book accessing and returning (BAR) operations. (**a**) The overall library robot structure. Inset: The robot body with a lifting rod fixed on it; (**b**) The robotic arm structure design for BAR operations.

To verify the applicability of the technique integration, we designed and fabricated a mobile robot prototype for BAR operations. Figure 1a presents the overall robot structure. As shown, such a robot consists mainly of two parts (i.e., the motion platform and the robot body). The motion platform, with both swerving and forward/backward moving functions, acts as the robot carrier. When given the motion command, it is able to navigate along the calculated trajectory to send the robot to a desired place. The robot body fixed on the motion platform is comprised of the inertial navigation system, the binocular vision system, and the QR code reader, as well as two robotic arms fixed on a lifting rod, and it is utilized to realize the BAR operations. The inertial navigation system helps identify the robot position and motion gestures in its operation process. The binocular vision system is responsible for rapid QR code region localization, dynamic digital map construction, autonomous robot P&N, as well as the obstacle identification and avoiding. Hence, when moving around in the library, the robot is able to update its given library digital map dynamically to eliminate the system operational errors, and also perform real-time route planning with the inertial navigation system, binocular vision system, and QR code reader working together. The two robotic arms, each of which is a serial manipulator with 4 degrees-of-freedom (DoFs), as shown in Figure 1b, are utilized to fulfill the BAR operations. Once reaching the target position, the robot would search for the target book with its QR code scanner, and determine the book position with its binocular vision system. The book positioning information finally drives the two robotic arms to fulfill the BAR operations. In this study, a two-arm structure is adopted, in which the two arms work cooperatively, with one arm being responsible for pulling the desired book out from the shelf, while the other is controlled to hold the book across its spine and put it to the basket fixed on the motion platform.

It is worth noting that, in this study, the size of the motion platform is customized to fit the space requirements of our lab. In practice, however, such a size could be changed and optimized according to the library requirements, such that the robot is able to navigate freely to the desired targets. Furthermore, as the heights of library bookshelves may also impact the library robot performance, the robot body is equipped with a lifting rod to adjust its height. As shown in the inset of Figure 1a, such a lifting rod is a mechanical structure with both rotating and rising functions. While the rotating function enables the robotic arms to access the books on both sides of a bookshelf corridor, the rising function helps the robot to reach the books that are high on the bookshelves. The serial design of the robotic arms themselves could also enable the robot to reach the books on bookshelves with certain heights.

In this study, two kinds of QR code labels, the book labels and the landmark labels, are generated with typical QR code generation software. The book labels containing detailed information of a book are utilized for robot BAR operations. Specifically, for each book, two identical book labels are used. One of such two book labels is pasted on the book spines for book accessing purpose, and the other one is pasted on the bookshelf at the corresponding position of the book, for book return purposes. In practice, since the page number varies from book to book, the size of the book labels should be different with both the page number and the QR code image scanner parameters taken into account. In this study, the size of QR code images were set to be 8 mm × 8 mm and 15 mm × 15 mm for books with small and large page numbers, respectively. As compared with the book labels, those landmark labels contain accurate 3D library coordinate spatial information, and they are pasted around the library for library map establishment and robot P&N accuracy improvements.

As compared with those book labels, the QR code landmarks contain accurate library 3D spatial coordinate information, and they are pasted around the library for library digital map updating and robot operational error corrections. When navigating around the library, the robot would identify the QR code region rapidly with its binocular vision system, and then access those landmarks with its QR code reader. The accurate library 3D spatial coordinate information contained within the QR code landmarks would help update the robot location information. Meanwhile, a closed-loop control system is also adopted to adjust the robot gestures such that it navigates along the calculated trajectory. In such a way, the robot operational errors accumulated within the robot P&N process would be eliminated, while the P&N accuracy could be significantly improved. All information of the books and the library digital map is maintained by a library management software system, and once the robot is given an operation command, it would navigate along the calculated trajectory to the desired target autonomously to perform the desired BAR operations.

### 2.2. Robot Binocular Vision System

#### 2.2.1. Robot Binocular Vision System Working Mechanism

The binocular vision system is one of the key components of the constructed robot. In this study, it is analogous to human binocular vision system, and is responsible for object identification and 3D co-ordinate calculations.

**Figure 2:**
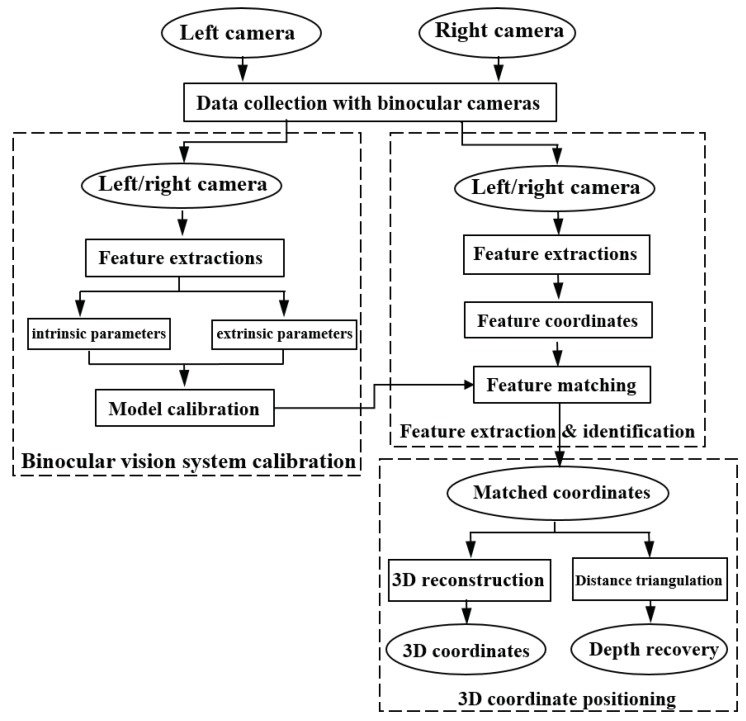
Working flow of the robot binocular vision system.

Figure 2 presents the main working flow of the robot binocular vision system. As seen, it consists of three main steps: Binocular vision system calibration, object feature extraction, and identification (O-FEI) and object 3D coordinate positioning. By acquiring a number of standard object images, the binocular vision system calibration process is used to determine the vision system property parameters. Those parameters include both intrinsic camera parameters (e.g., the camera focal lengths, camera optic center positions, and extrinsic camera parameters), like translational and rotational vectors. In contrast, the last two steps are responsible for the rapid QR code region identification, dynamic digital map construction, and autonomous P&N, as well as the obstacle identification and avoiding. Specifically, the O-FEI process helps the robot identify both QR code regions and obstacles in the robot trajectory. Once the QR code landmarks are identified, the robot would access such landmarks for the accurate coordinate information, which thus helps eliminate the robot operational errors and improve the P&N accuracy. While if obstacles are identified, the robot would update the library digital map and conduct real-time route re-planning to avoid such obstacles. The 3D coordinate positioning process is utilized to determine 3D coordinates of those objects and obstacles. With all those steps working together, the robot fulfills the rapid QR code regions identification, dynamic digital map construction, autonomous P&N, as well as the obstacle identification and avoiding functions.

Among those three steps, the binocular vision system calibration determines the overall operation accuracies of the robot. Below, we explain the binocular vision system calibration for camera parameter characterizations first, and then present the camera distortion error corrections.

#### 2.2.2. Binocular Vision System Calibration

**Figure 3:**
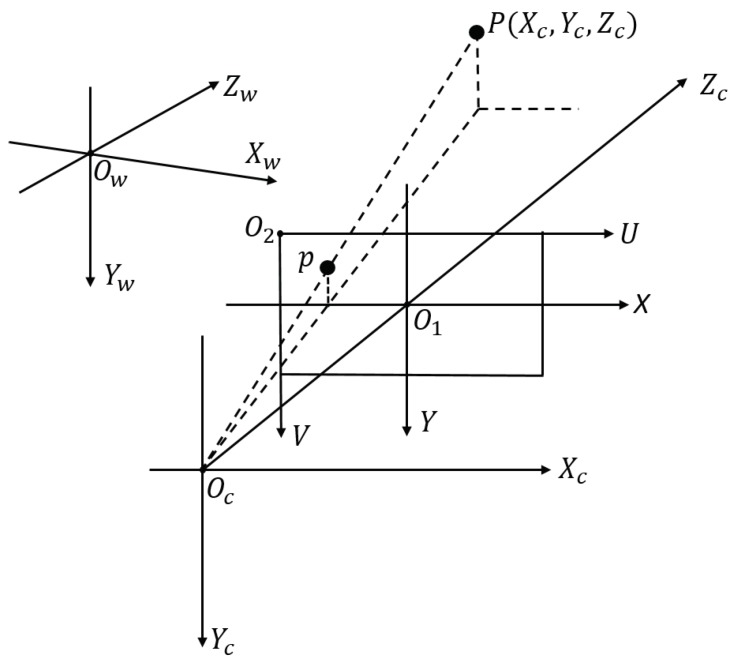
A pinhole system utilized for modeling a camera of the robot binocular vision system. It consists of four coordinate systems, wherein $O_{c}$ and $Z_{c}$ denote the optical center and optical axis of the camera, respectively.

A binocular vision system, consisting of two identical cameras, is adopted for the robot. Those two cameras are parallel to each other, and each of them could be modeled as a linear pinhole system, as shown in Figure 3. In such a model, there are four coordinate systems, of which $O_{w}-X_{w}Y_{w}Z_{w}$ is the free space coordinate system and $\;O_{c}-X_{c}Y_{c}Z_{c}$ is the camera coordinate system, while $O_{1}-XY$ and $O_{2}-UV$ are the image plane coordinate system and pixel plane 2D coordinate system, respectively. The free space coordinate system is utilized to denote the positions of both cameras and the other objects in the world.

Assume that the position of the coordinate system $O_{1}-XY$ origin $O_{1}$ in the coordinate system $O_{2}-UV$ is $\left(u_{0},v_{0}\right)$, then for any point (x,y) in $O_{1}-XY$, its position in $O_{2}-UV$ could be described by the homogeneous coordinate transformation matrix as shown in Equation (1),

(1) $$\left[\begin{array}{c} u\\ v\\ 1 \end{array}\right]=\left[\begin{array}{ccc} \frac{1}{dx} & 0 & u_{0}\\ 0 & \frac{1}{dy} & v_{0}\\ 0 & 0 & 1 \end{array}\right]\left[\begin{array}{c} x\\ y\\ 1 \end{array}\right],$$

where dx and dy are the physical size of each pixel in the *X* and *Y* axis directions, respectively.

Further, assume that $P(X_{c},\;Y_{c},\;Z_{c})$ is a point in the camera coordinate system $\;O_{c}-X_{c}Y_{c}Z_{c}$, and *p* is the image point of *P* within the image plane coordinate system $O_{1}-XY$. Hence, the coordinate (x,y) of *p* in $O_{1}-XY$ could be calculated as below,

(2) $$Z_{c}\left[\begin{array}{c} x\\ y\\ 1 \end{array}\right]=\left[\begin{array}{cccc} f & 0 & 0 & 0\\ 0 & f & 0 & 0\\ 0 & 0 & 0 & 1 \end{array}\right]\left[\begin{array}{c} X_{c}\\ Y_{c}\\ Z_{c}\\ 1 \end{array}\right],$$

where *f* is the focal length of the camera.

In practice, any point $\left(X_{w},\;Y_{w},\;Z_{w}\right)$ in the free space coordinate system $O_{w}-X_{w}Y_{w}Z_{w}$ could be mapped to the camera coordinate system $O_{c}-X_{c}Y_{c}Z_{c}$ using a translational vector **T** and rotational vector **R** as below,

(3) $$\left[\begin{array}{c} X_{c}\\ Y_{c}\\ Z_{c}\\ 1 \end{array}\right]=\left[\begin{array}{cc} R & T\\ 0^{T} & 1 \end{array}\right]\left[\begin{array}{c} X_{w}\\ Y_{w}\\ Z_{w}\\ 1 \end{array}\right],$$

where $0^{T}=\left[0\;0\;0\right]$, and $T=[t_{x},\;t_{y},\;t_{z}]$ is the position of the coordinate origin $O_{w}$ in the camera coordinate system $\;O_{c}-X_{c}Y_{c}Z_{c}$. Hence, the projection of any free space point $\left(X_{w},Y_{w},Z_{w}\right)$ in the pixel plane 2D coordinate system $O_{2}-UV$ could be described as follows,

(4) $$\begin{array}{cc} Z_{c}\left[\begin{array}{c} u\\ v\\ 1 \end{array}\right] & =\left[\begin{array}{ccc} \frac{1}{dx} & 0 & u_{0}\\ 0 & \frac{1}{dy} & v_{0}\\ 0 & 0 & 1 \end{array}\right]\left[\begin{array}{cccc} f & 0 & 0 & 0\\ 0 & f & 0 & 0\\ 0 & 0 & 1 & 0 \end{array}\right]\left[\begin{array}{cc} R & T\\ 0^{T} & 1 \end{array}\right]\left[\begin{array}{c} X_{w}\\ Y_{w}\\ Z_{w}\\ 1 \end{array}\right]\\ & =\left[\begin{array}{cccc} f_{x} & 0 & u_{0} & 0\\ 0 & f_{y} & v_{0} & 0\\ 0 & 0 & 1 & 0 \end{array}\right]\left[\begin{array}{cc} R & T\\ 0^{T} & 1 \end{array}\right]\left[\begin{array}{c} X_{w}\\ Y_{w}\\ Z_{w}\\ 1 \end{array}\right]\\ & =M_{1}M_{2}X_{W}=MX_{W}, \end{array}$$

where $f_{x}=\frac{f}{dx}$, $f_{y}=\frac{f}{dy}$, *M* is a 3×4 projection matrix, $M_{1}$ is a parameter determined by the camera properties $f_{x},\;f_{y},\;u_{0},\;v_{0}$, and $M_{2}$ is the position of camera in the free space coordinate system.

There are various calibration methods, such as manual calibration, Matlab tool box-based calibration, and OpenCV based self-calibration, for determining both the intrinsic and extrinsic parameters of a binocular vision system. In this study, the Matlab tool box-based calibration method is adopted for its higher accuracy and robustness as compared with the other methods. Once a number of standard target QR code images are collected, both intrinsic and extrinsic parameters of the system could be obtained and, finally, the mapping function between the free space coordinate system and image plane coordinate system can be obtained.

#### 2.2.3. Binocular Vision System Error Correction

Due to the practical manufacturing or assembly errors, however, the camera sensors are not always on its optical axis, especially for wide-angle cameras. In such a case, there may exist distortions in those out-off-center positions for the binocular vision cameras. Such distortions typically include the radial distortion, centrifugal distortion, and thin prism distortion, among which the radial distortion is regarded to be the factor contributing the most [24,25]. Since the radial distortion is typically regarded to be symmetrical relative to optical axis, two new parameters $\delta_{x}$ and $\delta_{y}$, as shown below, are introduced to correct the pinhole system,

(5) $$\left\{\begin{array}{cc} \delta_{x} & =k_{1}x\left(x^{2}+y^{2}\right)\\ \delta_{y} & =k_{2}y\left(x^{2}+y^{2}\right) \end{array}\right.,$$

where (x,y) is the coordinate of any imaging point, and $k_{1}$ and $k_{2}$ are distortion coefficients in the radial direction.

Therefore, the coordinates (x,y) of any point within linear pinhole system as illustrated by Equation (1) could be corrected as below,

(6) $$\left\{\begin{array}{cc} \bar{x} & =x+\delta_{x}=x\left(1+k_{1}r^{2}\right)\\ \bar{y} & =y+\delta_{y}=y\left(1+k_{2}r^{2}\right) \end{array}\right.,$$

where $r^{2}=x^{2}+y^{2}$. The equation above indicates that the binocular vision system distortions in the directions of *x* and *y* are proportional to the square of the radius.

To calibrate the binocular vision system while correcting the radial distortion, a two-step iteration algorithm is adopted to separate the intrinsic and extrinsic camera parameters from those distortion ones, and process them separately. The linear transformation (LT) or perspective transformation matrix (PTM) method could be adopted to obtain the camera parameters first and, then, those obtained parameters are utilized as initial values for the second step iteration to obtain the optimal solutions. In such a way, the influences of both the initial values and distortion coefficients to binocular vision system calibrations are considered and minimized. In this study, the LT method is adopted to obtain the camera parameters.

## 3. Library Robot P & N and BAR Mechanism

Compared to the structure design, the mechanism implementation for robot P&N and BAR operations is more complicated. This is because such a mechanism consists of many other functional sub-mechanisms; that is, the real-time robot route planning algorithm, the QR-code feature extraction and identification (QR-FEI) algorithm, and the QR-code based positioning accuracy correction algorithm, as well as the robot BAR operation algorithm. Each of those mechanisms will be described below.

### 3.1. Real-Time Robot Route Planning

Once given the target, the library robot would start to navigate along the calculated shortest route to it. In practice, however, as the obstacles in libraries are usually distributed randomly and sometimes may even appear suddenly and block the robot trajectory, the robot has to perform real-time route planning. Such a function helps the robot find the shortest path to the target, and also avoid those obstacles. In this study, the improved $D^{*}$ Lite algorithm, which supports incremental re-planning, is adopted for real-time robot route planning [26,27,28].

**Figure 4:**
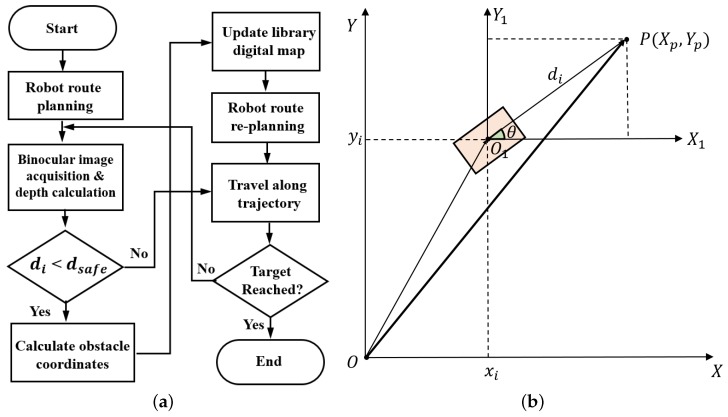
(**a**) Binocular vision system imaging based robot real-time route planning mechanism. (**b**) A schematic image showing the 2D robot and library base coordinate systems.

Figure 4a presents the binocular vision system imaging-based robot real-time route planning mechanism. When navigating to the target, the robot would acquire images along its trajectory periodically with the binocular vision system, and then calculate a depth value $d_{i}$ immediately based upon its binocular vision system parallax. Such a $d_{i}$ value is finally compared to a predefined threshold $d_{safe}$ to determine whether there is any obstacle along the route or not. When $d_{i}$ is larger than $d_{safe}$, it is assume that there is no obstacle and the robot keeps moving along its trajectory, while if $d_{i}$ is smaller than $d_{safe}$, it is assumed that obstacles are detected in the robot trajectory, and the shortest robot trajectory should be recalculated. In such a case, position information of the detected obstacles in library base coordinate system has to be calculated and remapped back to the library grid map.

Assume that the library base coordinate system in 2D plane is O−XY and the robot 2D coordinate system is $O_{1}-X_{1}Y_{1}$, while the position of an obstacle *P* in $O_{1}-X_{1}Y_{1}$ is $P(X_{p},Y_{p})$, as shown in Figure 4b. Once a depth value $d_{i}$ is smaller than $d_{safe}$ for the robot at a position $\left(x_{i},y_{i},\theta_{i}\right)$, the obstacle position *P* in the base coordinate system (i.e., $\vec{OP}$), has to be calculated as follows,

(7) $$\left(X_{p},Y_{p}\right)=\vec{OP}=\vec{OO_{1}}+\vec{O_{1}P}=\left(x_{i}+d_{i}\cos\theta_{i},\;y_{i}+d_{i}\sin\theta_{i}\right),$$

where $\vec{OO_{1}}=\left(x_{i},\;y_{i}\right)$ and $\vec{O_{1}P}=\left(d_{i}\cos\theta_{i},\;d_{i}\sin\theta_{i}\right)$ as shown in Figure 4b.

The position of *P* in library grid map could be obtained using the equation below,

(8) $$\left(x\left[\;\right],\;y\left[\;\right]\right)=F\left(x_{p},\;y_{p}\right),$$

where (x[],y[]) are arrays, while F(x,y) is the function utilized to remap the position (x,y) from base coordinate to grid map and is given by Equation (4) in Section 2.2.

Therefore, once (x[],y[]) is obtained, the elements (x[],y[]) are updated with (x[],y[])=1 to denote that those positions in the library grid map are occupied by obstacles, and then the improved $D^{*}$ Lite algorithm is adopted to re-compute a shortest route for the robot. The main working steps of such a route planning mechanism are shown in Algorithm 1, wherein the Dijkstra’s shortest path algorithm could be modified to compute the shortest path between any two positions in the grid map. To achieve a balance between the computation load and the navigation accuracy, the camera image acquisition period was set to be 50 ms in this study. Such a period guarantees that once an obstacle is detected, the robot is able to perform real-time re-routing in time to avoid it.

**Algorithm 1** The improved $D^{*}$ Lite algorithm for real-time robot route planning.**Input:** Target location **$S_{goal}$**, Robot starting point **$S_{start}$**, Library grid map ***M***.**Output:** Planned trajectory **$T_{r}$** for library robot. 1.8 1: Initialization. $d_{safe}$; $S_{last}=S_{start}$; 2: **$T_{r}$** = Computeshortestpath(M, $S_{start}$, $S_{goal}$); 3: **while**
$S_{last}\neq S_{start}$
**do** 4:     $S_{start}=\arg\min_{s\prime \in Succ(S_{start})}\left(c\left(S_{start},s\prime \right)+g\left(s\prime \right)\right)$; 5:     Move to $S_{start}$; 6:     Scan library grid map ***M*** for changed edge costs; 7:     **if** any edge costs changed **then** 8:          update grid map *M* according Equation (8); 9:          $k_{m}=k_{m}+h\left(S_{last},S_{start}\right)$; 10:         $S_{last}=S_{start}$; 11:         **for** all directed edges (u,v) with changed edge costs **do** 12:             updated the edge cost c(u,v); 13:             **$T_{r}$** = Computeshortestpath(M, $S_{start}$, $S_{goal}$); 14:         **end for** 15:     **end if** 16: **end while** 17: Return robot trajectory **$T_{r}$**.

### 3.2. The QR-FEI Algorithm

**Figure 5:**
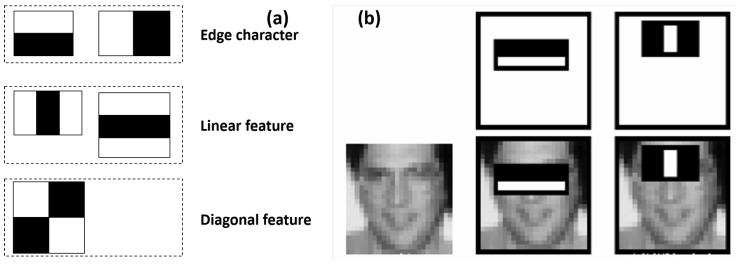
Haar features for object identification. (**a**) Typical Haar-like features. (**b**) Denoting a face utilizing the Haar-like feature.

The Haar-like feature, which is group of characteristics consisting mainly of the edge, line, point, and diagonal features, as shown in Figure 5a [29], was adopted for the QR code identifications in this study. To denote an object, such a feature is typically made up of at least two rectangles, as shown in Figure 5b, and its value is the difference between the sums of the pixel values within the black and white rectangles.

Owing to its reduced computational complexity and improved operating speed, the Viola-Jones integral graph algorithm [30] was adopted for QR code Haar-like feature extraction in this study. Hence, for an image *i*, its integral graph ii at location of a point pixel(x,y) contains the sum of the pixel values above and left of this pixel (x,y) (i.e., the upper left corner of pixel(x,y)), and its feature value could be calculated as shown in Equation (9).

(9) $$ii\left(x,y\right)=\sum_{x^{\prime }\leq x}\sum_{y^{\prime }\leq y}i\left(x^{\prime },y^{\prime }\right)$$

When detecting the target QR codes, the image is scanned by a sub-window that contains a specific Haar-like feature as shown in Figure 5b. Based on each Haar-like feature, $h_{i}\left(x\right)$, the corresponding classifier $f_{i}\left(x\right)$ is given by Equation (10) below,

(10) $$f_{i}\left(x\right)=\left\{\begin{array}{cc} 1 & p_{i}h_{i}\left(x\right)<p_{i}\theta_{i}\\ -1 & \mathrm{otherwise}. \end{array}\right.,$$

where *x* is the sample within the sub-window, $h_{i}\left(x\right)$ is the value of the *i*-th Haar feature $h_{i}$ of the sample *x*, $p_{i}\in \left[-1,\;1\right]$ is the symbol of classification direction, $f_{i}$ is the classifier made up of features $h_{i}$, and $\theta_{i}$ is the threshold of classifier $f_{i}$.

**Figure 6:**
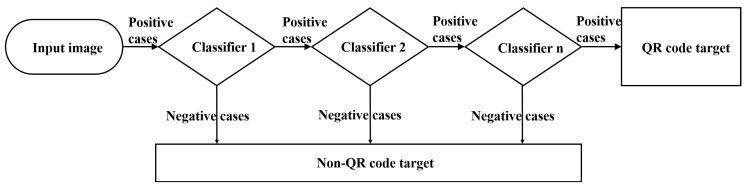
Cascaded classification model for quick response (QR)-code feature extraction.

In this study, the stable self-adaptive Adaboost learning algorithm [31] was adopted to improve the object detection accuracy. The Adaboost algorithm is based on a cascade classification model, as shown in Figure 6, wherein a series of weak classifiers are cascaded together to improve the detection accuracy iteratively until the desired accuracy is achieved. The main purpose of such a strategy is to find an optimal weak classifier to achieve the lowest misclassification rate. Owing to the selective properties of those multi-level weak classifiers, the selection efficiency of the Adaboost learning algorithm was largely improved.

### 3.3. QR Code Based Positioning Accuracy Correction Algorithm

To improve the robot P&N accuracy, the robot operational errors accumulated in its navigation process must be eliminated. In this paper, a parallax processing-based 3D reconstruction algorithm was proposed, for such a purpose.

**Figure 7:**
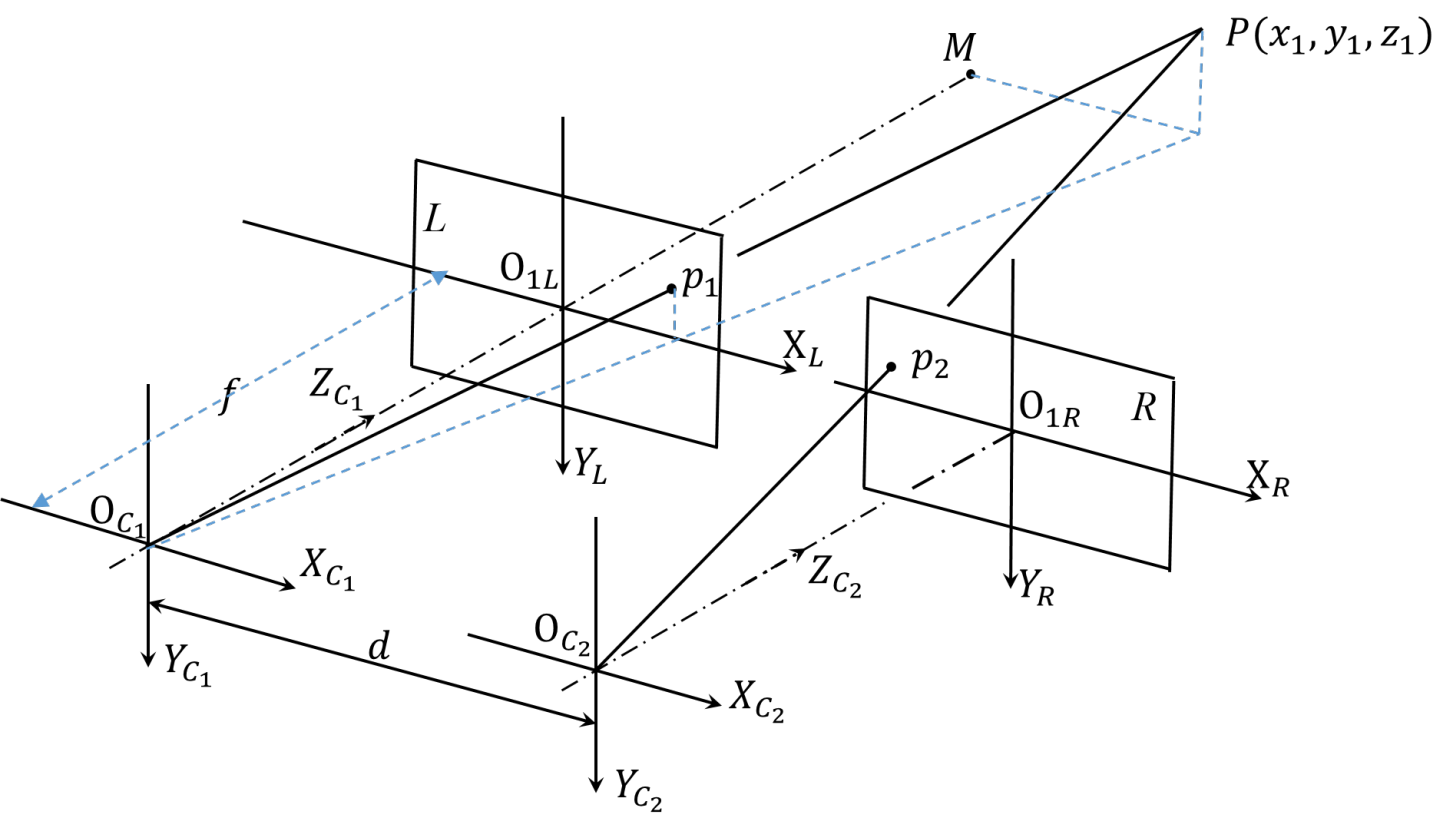
Schematic for 3D reconstruction for a point *P* in the binocular vision system.

Figure 7 presents a schematic of the robot binocular vision system, wherein two identical cameras are utilized. Suppose that $O_{C_{1}}$ and $O_{C_{2}}$ are optical centers of the two cameras, *d* is the distance between those two optical centers, and *f* is the focal length of the camera. Further, assume that the coordinates of an object *P* within the left and right camera coordinate systems, which are denoted to be $O_{C_{1}}-X_{C_{1}}Y_{C_{1}}Z_{C_{1}}$ and $O_{C_{2}}-X_{C_{2}}Y_{C_{2}}Z_{C_{2}}$, are $\left(x_{1},y_{1},z_{1}\right)$ and $\left(x_{2},y_{2},z_{2}\right)$, while its location in the two images acquired by the two cameras, is $\left(u_{1},v_{1}\right)$ and $\left(u_{2},v_{2}\right)$, respectively. Hence, Equation (11) can be obtained,

(11) $$\left\{\begin{array}{cc} u_{1}-u_{0} & =f_{x}\frac{x_{1}}{z_{1}}\\ v_{1}-v_{0} & =f_{y}\frac{y_{1}}{z_{1}}\\ u_{2}-u_{0} & =f_{x}\frac{x_{2}}{z_{1}}=f\frac{x_{1}-d}{z_{1}} \end{array}\right..$$

While the position of point *P* within the 3D camera system could be obtained, as shown in Equation (12),

(12) $$\left\{\begin{array}{cc} X=x_{1} & =\frac{d(u_{1}-u_{0})}{u_{1}-u_{2}}\\ Y=y_{1} & =\frac{df_{x}\left(v_{1}-v_{0}\right)}{f_{y}\left(u_{1}-u_{2}\right)}\\ Z=z_{1} & =\frac{df_{x}}{u_{1}-u_{2}} \end{array}\right.,$$

where $u_{0},\;v_{0},\;f_{x}$, and $f_{y}$ are intrinsic parameters of the cameras. They are the same as those in Equation (4), and are determined in the camera calibration process.

Therefore, once a number of QR code landmarks are identified, both the landmark position information within the 3D camera system and the relative position between the robot and the landmarks can be determined. Finally, the robot position within the free space coordinate system can also be obtained. In such a way, the robot positioning error accumulated within the robot navigation process could be eliminated. Meanwhile, both the robot gestures and its trajectory to the target could be adjusted.

### 3.4. Robot BAR Operation Algorithm

**Figure 8:**
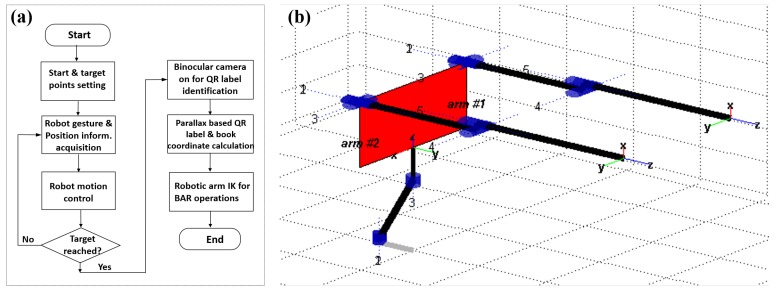
(**a**) The binocular vision based robot BAR operation control mechanism. (**b**) The modified- DH convention based link diagram for the robotic arms.

The robot BAR operations were implemented with its two robotic arms, and the binocular vision based robot BAR operation control mechanism is shown in Figure 8a. Specifically, once the robot reaches the target along the calculated trajectory, its binocular cameras are turned on to search for the target QR code labels, and then the QR-code shape and feature point detection mechanism is utilized to extract the detailed book information (i.e., the position and orientation information within 3D free space coordinate system). Such acquired book coordinate information would finally drive the two robotic arms to work cooperatively to fulfill the BAR operations.

To fulfill such a BAR function, both the robotic kinematics [32] and inverse kinematics [33] of the library robot’s two robotic arms have to be determined. The robotic kinematics is used to calculate the final position of the robot accessing hand when given the operations of each joint structure, while the inverse kinematics (IK) is used to determine the operations of each robot joint structure once given the final position of the accessing hands. In this study, the robotic 4-DoFs manipulator, shown in Figure 1b, was modeled as a serial joint link structure based upon the modified-DH convention [34]. The link diagram for such a manipulator is illustrated in Figure 8b, and its modified DH parameters are shown in Table 1. Furthermore, since the relation between i−1-th and *i*-th frame could be expressed by Equation (13),

(13) $${}_{i}^{i-1}T=\left[\begin{array}{cccc} c\theta_{i} & -s\theta_{i} & 0 & a_{i-1}\\ s\theta_{i}c\alpha_{i-1} & c\theta_{i}c\alpha_{i-1} & -s\alpha_{i-1} & -s\alpha_{i-1}d_{i}\\ s\theta_{i}s\alpha_{i-1} & c\theta_{i}s\alpha_{i-1} & c\alpha_{i-1} & c\alpha_{i-1}d_{i}\\ 0 & 0 & 0 & 1 \end{array}\right],$$

where *c* and *s* correspond to the cos and sin functions, $a_{i}$ and $\alpha_{i}$ are link length and torsion angle along *a* axis for frame i−1, respectively, and $d_{i}$ and $\theta_{i}$ are the link offset and rotation angle along *z* axis for frame i−1, respectively. Then, the robotic kinematics of the two robotic arms could be expressed as shown in Equation (14):

(14) $${}_{4}^{0}T={}_{1}^{0}T\;{}_{2}^{1}T\;{}_{3}^{2}T\;{}_{4}^{3}T=\left[\begin{array}{cccc} n_{x} & o_{x} & a_{x} & p_{x}\\ n_{y} & o_{y} & a_{y} & p{}_{y}\\ n_{z} & o_{z} & a_{z} & p_{z}\\ 0 & 0 & 0 & 1 \end{array}\right],$$

where the 3×3 matrix in the left upper corner describes the robot accessing hand gestures, while ${\left[p_{x},p_{y},p_{z}\right]}^{T}$ denotes the accessing hand position within the spatial coordinate.

Further introducing Equation (13) into Equation (14), we have the parameters as follows,

(15) $$\left\{\begin{array}{c} n_{x}=s\theta_{1}s\theta_{4}+c\theta_{4}c\theta_{23}c\theta_{1}\\ n_{y}=-c\theta_{1}s\theta_{4}+c\theta_{4}c\theta_{23}s\theta_{1}\\ n_{z}=-s\theta_{23}c\theta_{4}\\ o_{x}=s\theta_{1}c\theta_{4}-s\theta_{4}c\theta_{23}c\theta_{1}\\ o_{y}=-c\theta_{1}c\theta_{4}-s\theta_{4}c\theta_{23}s\theta_{1}\\ o_{z}=s\theta_{23}s\theta_{4}\\ a_{x}=-s\theta_{23}c\theta_{1}\\ a_{y}=-s\theta_{23}s\theta_{1}\\ a_{z}=-c\theta_{23}\\ p_{x}=c\theta_{1}\left(a_{3}c\theta_{23}+a_{2}c\theta_{2}\right)\\ p_{y}=s\theta_{1}\left(a_{3}c\theta_{23}+a_{2}c\theta_{2}\right)\\ p_{z}=-a_{3}s\theta_{23}-a_{2}s\theta_{2} \end{array}\right.,$$

where $\theta_{23}=\theta_{2}+\theta_{3}$.

In order to characterize the IK model for the library robot, those joint link parameters $\theta_{1}$ to $\theta_{4}$ have to be determined. By utilizing Equation (15), $\theta_{1}$ could be calculated with $p_{x}$ and $p_{y}$ (i.e., $\theta_{1}=\arctan\frac{p_{y}}{p_{x}}$). Also, by using $o_{z}=s\theta_{23}s\theta_{4}$ and $n_{z}=-s\theta_{23}c\theta_{4}$, $\theta_{4}$ could be obtained as $\theta_{4}=\arctan\left(\frac{o_{z}}{n_{z}}\right)$ only when $s\theta_{23}\neq 0$ (when $s\theta_{23}=0$, the joint axis is at a singular point, and the IK model can not be solved). The parameter $\theta_{23}$ could be obtained with $o_{z}$ and $a_{z}$ as $\theta_{23}=\arctan\left(\frac{o_{z}}{-a_{z}s\theta_{4}}\right)$ when $s\theta_{4}\neq 0$. By utilizing $p_{y}$ and $p_{z}$, the parameter $\theta_{2}$ could also be obtained with Equation (16),

(16) $$\left\{\;\begin{array}{cc} \theta_{2} & =\arctan\frac{s\theta_{2}}{c\theta_{2}}\\ s\theta_{2} & =\frac{-(p_{s}+d_{4}s\theta_{23})}{d_{3}}\\ c\theta_{2} & =\frac{\left(p_{y}/s\theta_{1}-d_{4}c\theta_{23}\right)}{d_{3}} \end{array}\right.,$$

while the parameter $\theta_{3}$ is determined as $\theta_{3}=\theta_{23}-\theta_{2}$.

Finally, the IK model for the two 4-DoFs robotic arms can described as below,

(17) $$\left\{\begin{array}{cc} \theta_{1} & =\arctan(p_{y}/p_{x})\\ \theta_{4} & =\arctan(o_{z}/n_{z})\\ \theta_{23} & =\arctan(o_{z}/-a_{z}s\theta_{4})\\ s\theta_{2} & =-\left(p_{z}+d_{4}s\theta_{23}\right)/d_{3}\\ c\theta_{2} & =\left(p_{y}/s\theta_{1}-d_{4}c\theta_{23}\right)/d_{3}\\ \theta_{2} & =\arctan(s\theta_{2}/c\theta_{2})\\ \theta_{3} & =\theta_{23}-\theta_{2} \end{array}\right..$$

Therefore, once the final position of the accessing hand is determined (i.e., the center point of a bookmark label is accessed by the robot QR code label), such an IK model could be adopted to determine the operation gestures for those two robotic arms to fulfil the desired BAR operations. In this study, since the mechanical structure of those robotic arms are relatively simple, the algebraic method was adopted to obtain the IK model solutions.

While for the book returning process, the procedure of robot P&N is the exactly the same as that of the book accessing process. The only difference between the returning and accessing processes is to find the desired position on book shelf to put the book back. To do so, the robot would scan the book labels on the bookshelves first, and then search for the label IDs such that the ID of the book to be returned falls in between. In such a way, once the ID position is determined, both the robot motion displacement and its arm gestures could be determined. One of the robotic arm is controlled to take the book from the basket, while the other arm is driven to insert the accessing hand into two books that were found to obtain a space for the book. Finally, the book would be placed into this space to fulfill the returning operation.

## 4. Simulation and Experimental Verification

Both simulations and experiments were conducted with the constructed robot to verify the effectiveness of the proposed technology combination.

### 4.1. Binocular Vision System Calibration

**Figure 9:**
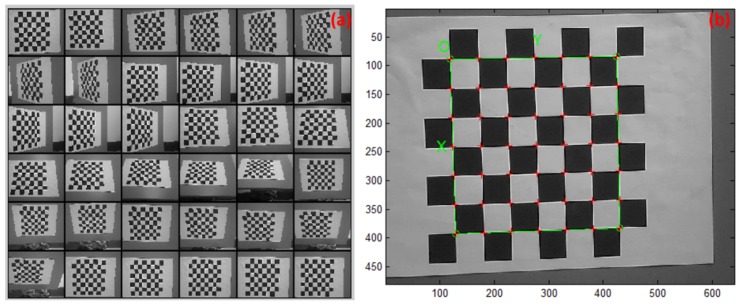
Library robot binocular vision system calibration. (**a**) The original QR code images utilized for calibration. (**b**) The corner-point images extracted from the original QR code images.

Several groups of chessboard-like QR code images acquired with the binocular vision system were utilized. The Matlab calibration toolbox was adopted for calibration processing, due to its higher accuracy and robustness. A number of QR code images, as shown in Figure 9a, were imported to the Matlab toolbox first and, then, the corner information for each image was extracted to generate the chessboard-like corner-information image, as shown in Figure 9b. Finally, the binocular vision system calibration was conducted with the corner-point information of all images being extracted.

**Figure 10:**
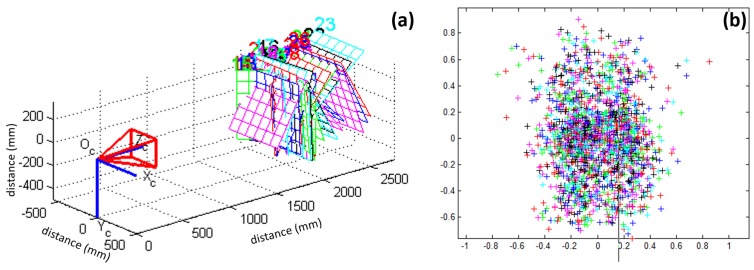
Calibrated camera system and its mapping effects. (**a**) The left camera model of the binocular vision system. (**b**) The projection error of the left camera system.

In the experiments, the system calibration was conducted for the left and right cameras to obtain the intrinsic parameters sequentially. Simulations on such a binocular vision system were also conducted to test the system mapping errors first, before implementation. The calibrated system model and the system mapping errors for the left camera are shown in Figure 10a,b, respectively, while the intrinsic parameters for the left camera are shown in (the left column of) Table 2. The same procedure was also conducted to calibrate the right camera of the binocular system, and the obtained intrinsic parameters are also shown in (the right column of) Table 2.

**Figure 11:**
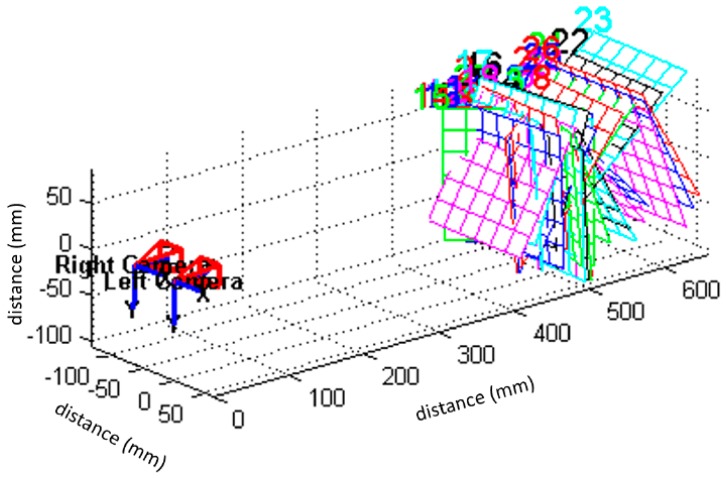
Simulated binocular vision system model configuration.

Finally, the optimal extrinsic parameters of the binocular vision system could also be obtained with the intrinsic parameters of both left and right cameras taken into account. The extrinsic parameters (i.e., rotation vector **R** and translation vector **T**), for such a binocular system, are shown in Table 3, while the simulated binocular vision system configuration is presented in Figure 11. The commercially-available binocular vision system (RER-720P2CAM-45, RERVISION Inc., China), as shown in Figure 1, was configured according to the parameters obtained from the simulated system model.

### 4.2. QR Code Feature Extraction and Identification

**Figure 12:**
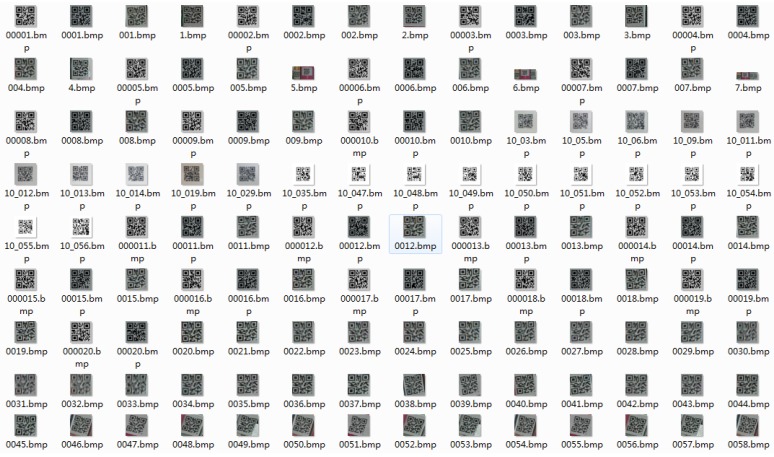
Part of the positive QR code image samples.

**Figure 13:**
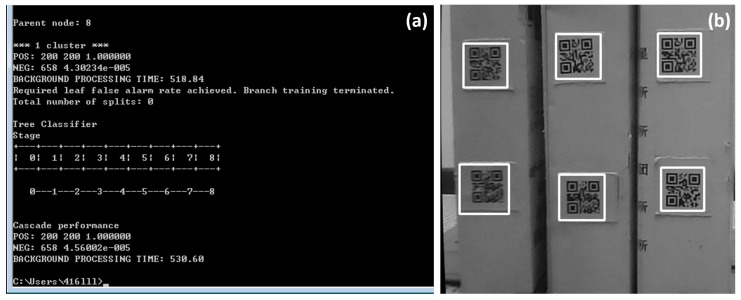
(**a**) QR code image classification results, based on the Haar feature; (**b**) QR code identification results.

The QR code sample generation and training were implemented with the tools provided by Opencv vision library. In this study, 200 positive samples, together with 658 negative samples, were collected. Those positive samples contained only the QR codes, while the negative ones contained no QR codes. Figure 12 presents some representative positive sample images, each of which was normalized to be 50 pixels ×50 pixels, and Figure 13a presents the generated results. Once sample generation and training were completed, Opencv generated an XML file that helped to realize the QR code identification operations. Figure 13b presents an example of the detection results for some desired QR code images, wherein the image areas that contained the QR codes are marked with white rectangular boxes.

### 4.3. QR Code Based Positioning

**Figure 14:**
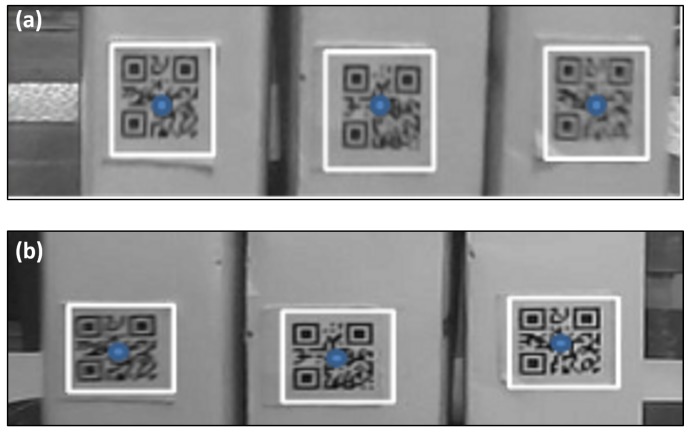
A set of QR code images acquired by the robot binocular vision system. (**a**) QR images obtained by left-hand side camera. (**b**) QR images acquired by the right-hand side camera.

The QR-code landmarks were utilized to eliminate the robot operational errors accumulated within its P&N process. Specifically, the binocular vision system was utilized to locate the QR-code region first. To obtain 3D coordinates of the QR code in the free space coordinate system, images of the same target QR code were acquired with the right and left hand-side cameras simultaneously. Figure 14 presents an example of a target QR code image region acquired with the binocular vision system. When those QR code images were acquired, they would be marked by white rectangular boxes; meanwhile, the center points of those rectangles would be calculated and marked. Those blue dots within the QR code images, as shown in Figure 14, denote the calculated center points of the recognized white rectangular boxes. Finally, those rectangular boxes were rearranged, such that their sequences of arrangement were the same in both right- and left-hand side cameras. The Equation (12) was utilized to calculate the 3D coordinates of the QR code images.

Similarly, the mobile robot would also access QR-code book labels to obtain the accurate 3D coordinate information of the target book, and then drive the two robotic arms to perform the desired BAR operations. To do so, both the position and orientation of the target book have to be determined. In this study, such information was obtained from those images acquired by the binocular vision system using the QR-FEI algorithm. Specifically, the book position information was obtained by calculating the pixel plane coordinates of the QR-code image center point first and, then, remapping those pixel plane coordinates back to the 3D coordinates of the free space, using Equation (12). In contrast, the orientation information of the book was determined by the slope of the QR-code image rectangular shape outline. Such an outline slope information together with the position information finally decide the operations of the robotic arms. In case of book misplacement (i.e., books are placed with an arbitrary orientation on the shelf), the slope of the QR-code image rectangular shape outline could also be utilized. For example, the QR-code could designed with a rectangular shape, and thus, slope of the detected rectangular QR-code shape outline could be utilized to determine the orientation of the book. Additional shape marks without any information on the QR code images (e.g., a horizontal or vertical line at certain position of the QR code image), could also be a candidate solution to address such a misplacement issue. For simplicity, without loss of generality, in this study, we assume that the QR-code slope was 90° (i.e., all the books are vertical to the shelves with no QR-code occlusions and misplacements).

Table 4 presents the 3D coordinates of a certain position calculated five times. Such results showed that among those five sets of data, the largest error was around 3.7 mm from the *Z*-axis, which was within the range for the QR code reader to scan and access. Such positioning results also convincingly demonstrate that the accuracy of our proposed mechanism is able to meet the requirements for practical library robot P&N operations.

### 4.4. Robot Trajectory Planning

#### 4.4.1. Real-Time Robot Trajectory Planning

**Figure 15:**
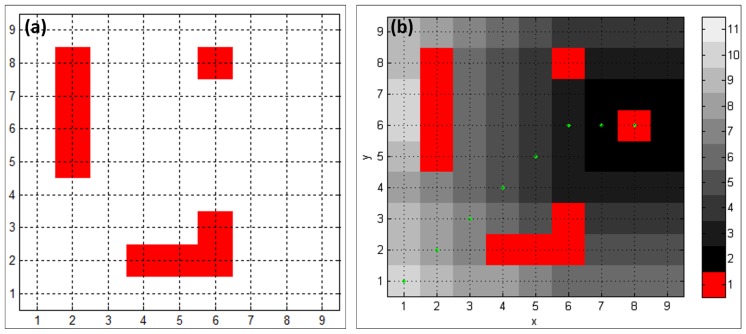
The library grid map utilized for robot route planning experiments. (**a**) A 9×9 grid map established for the lab space; (**b**) The robot route established with $D^{*}$ Lite algorithm.

Experiments were conducted within a lab with floor dimensions of 4.20 m × 4.20 m, while the size of the mobile robot was 0.45 m in length and 0.35 m in width. To establish the library digital map, the lab space was divided into a grid with a resolution of 0.45 m × 0.45 m and, therefore, a 9 × 9 grid map, as shown in Figure 15a, was obtained. In Figure 15a, the red portion indicates that such areas were occupied by the obstacles. As for route planning testing, as shown in Figure 15a, we set the target and the starting point to be g=(8,6) and s=(1,1) in the grid map, respectively. The $D^{*}$ Lite algorithm was adopted for the robot trajectory calculations, and thus, the trajectory denoted by the green dots in Figure 15b was established.

**Figure 16:**
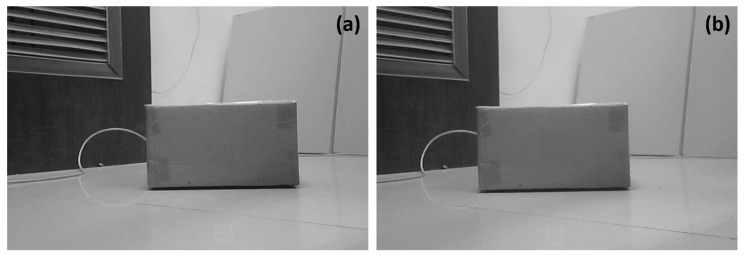
Two images of an obstacles identified acquired by robot binocular vision system for obstacle position calculation. (**a**) An obstacle image acquired by the left hand-side camera; (**b**) An obstacle image acquired by right hand-side camera.

To implement the robot autonomous route re-planning function with the $D^{*}$ Lite algorithm, a safe depth threshold $d_{safe}$ had to be calculated to determine the robot’s motion. In this study, such a depth threshold $d_{safe}$ was set to be the hypotenuse length of two grids (i.e., $d_{safe}=\sqrt{2}\times 0.45\times 2=1.27\;m=1270\;\mathrm{mm}$). When navigating along the route to its target, the robot would calculate a depth $d_{i}$ based on its the binocular vision system parallax, and compare $d_{i}$ and $d_{safe}$ to determine its motion status. Meanwhile, the position of the robot in the lab spatial map system was also characterized using the camera positioning system and the electronic compass equipped on the robot. Figure 16a,b present the two images of an obstacle that were acquired by the left and right hand-side cameras of the robot binocular vision system, respectively. Based on the parallax generated from the binocular vision system, the depth value $d_{i}$ and the position $\left(X_{p},Y_{p}\right)$ in the base coordinate system of such an obstacle was calculated to be 1264.7mm and (2715.85mm,2695.16mm), respectively. In addition, the robot’s position was calculated to be (1815.2mm,1807.3mm) in the library digital map, with an angle of 44.59°.

**Figure 17:**
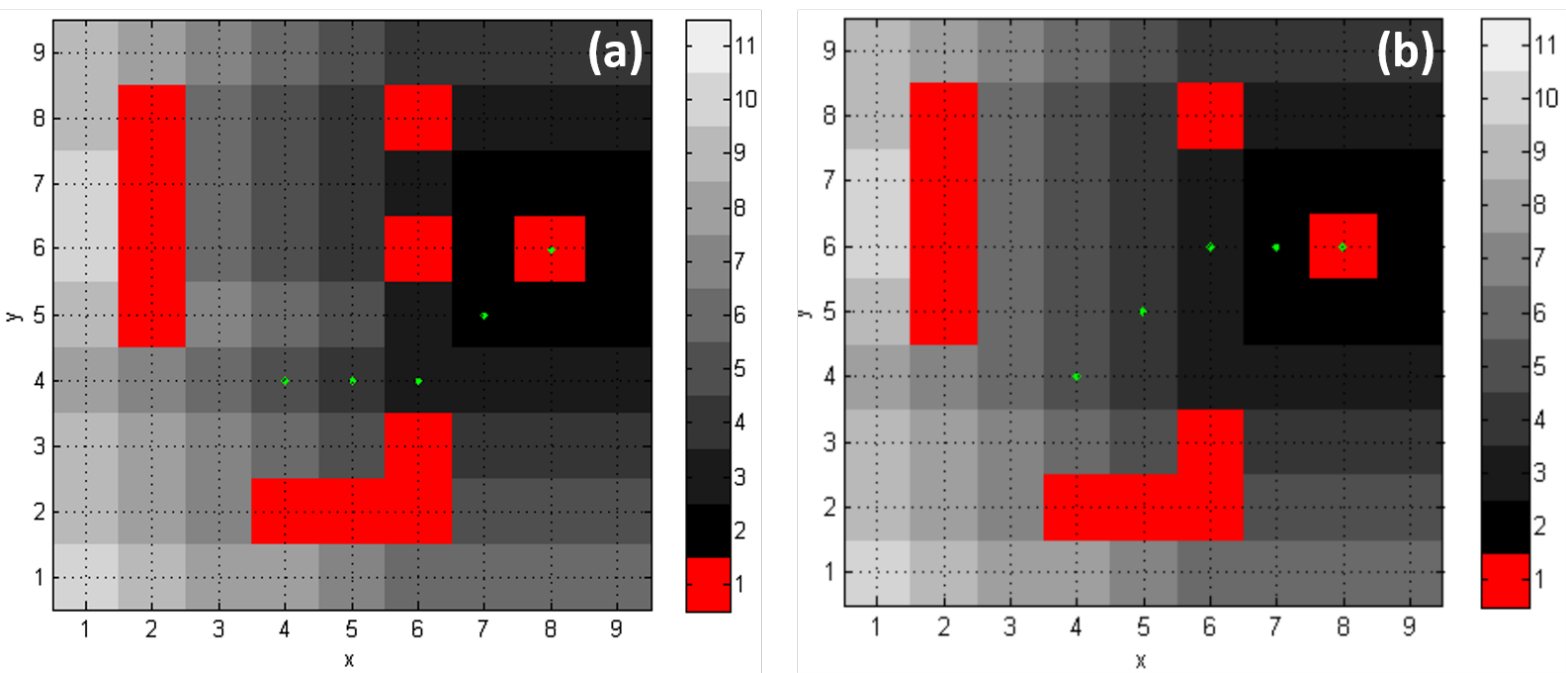
Re-planned trajectory route compared to the original one. (**a**) The re-planned robot trajectory after an obstacle was detected at g(6,6); (**b**) The original robot trajectory route.

Since the depth value $d_{i}=1264.7$ mm was smaller than the depth threshold $d_{safe}=1270$ mm (i.e., $d_{i}<d_{safe}$), the obstacle was marked as detected. The obstacle spatial position $\left(X_{p},Y_{p}\right)$ in the base coordinate system should be re-mapped to the grid map with the re-mapping function, given in Equation (8). Therefore, the obstacle coordinates in the grid map were calculated to be (6,6). When performing route re-planning function for the robot, the obtained obstacle grid coordinate was set to be g(6,6)=1, and the current robot location coordinate was set to be its new starting point. The re-planned new robot route is shown in Figure 17a, wherein the new route avoids the obstacle at g(6,6), as compared to the original route, shown in Figure 17b.

**Figure 18:**
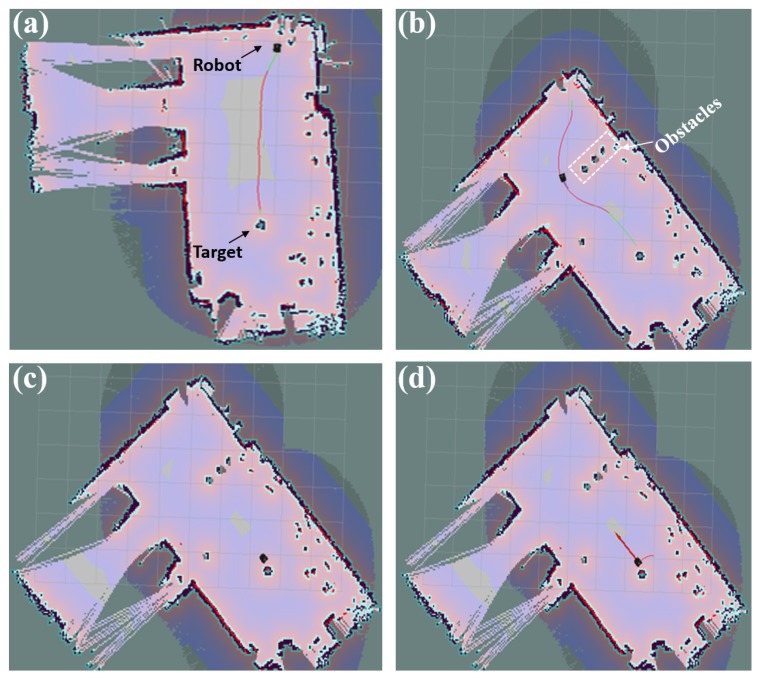
Experimental verification of the robot real-time route re-planning function with the binocular vision system using the X–Y plane projection map. (**a**) A shortest path route calculated on the initial digital map between the robot and target; (**b**) A new path calculated from the new robot location to target once obstacles were identified during its navigation process; (**c**) Robot reaches the target with the obstacles avoided; (**d**) Robot route planning process for returning back to the starting point.

To further simulate the dynamic environment of real libraries, we also conducted experiments to test the robot’s real-time route re-planning function by placing obstacles to block the robot trajectory during its moving process. Figure 18 presents the X–Y plane projection of a robot obstacle identification and avoiding process in the free space 3D coordinate system. At the beginning, as seen in Figure 18a, both the robot and target were placed at a random position, and robot calculated a shortest path (the red line on the map in Figure 18a), from the robot’s starting point to the target. During the robot navigating process, however, obstacles were placed to block its trajectory. In such a case, the robot identified such obstacles with its binocular vision system and then updated the digital map immediately. Meanwhile, it re-calculated a shortest path from its current location to the target, as shown in Figure 18b. Once it reached the target to fulfill its BAR operations, as shown in Figure 18c, the robot finally calculated another new route from its target to its starting point. Figure 18d presents the robot navigating from the target back to its starting point. We conducted such an experiment 10 times in our lab, and achieved satisfactory results with no obstacles touched. Such results demonstrated that both the binocular vision and QR-code identification integrated technology, as well as the constructed robot, are robust.

#### 4.4.2. Binocular Vision System Function for Robot Trajectory Planning

The binocular vision system plays a critical role in the robot dynamic route planning process, as it helps to detect and avoid obstacles in time. However, if a single global path is calculated for the robot, the robot may not be able to detect and avoid those obstacles in time, especially for those obstacles appearing in the robot navigation process, which, thus, could raise the trajectory planning failing rate. To verify the effectiveness of the binocular vision system, experiments were also conducted to compare robot route planning performances, with and without the binocular vision system when a single global path was calculated.

In the experiments, the robot starting points, targets, and obstacles were placed in the lab area randomly, and each experiment was conducted 10 times. Table 5 presents a comparison of the robot route planning operations, with and without the binocular vision system. Results show that, without the binocular vision system, the robot hit the obstacle five times and also failed two times in reaching the target, while, with such a camera system, the P&N accuracy was largely improved, and all the 10 tests were successfully finished without hitting the obstacle. Specifically, for the time utilized for successful target accessing, it took 321 s for the eight times with a single global route calculated, while it took only 246 s for the 10 successful times with a binocular vision system adopted. The average time spent for a successful route planning operation was ∼40.1 s and ∼24.6 s for the two cases, respectively (i.e., the average operation time was reduced by ∼38.4% with the binocular vision system). Such results demonstrate that the binocular system helps to improve the robot positioning accuracy largely, and also significantly saves route planning time; as compared to the global planing method.

### 4.5. Robot BAR Operation Verification

Experiments were conducted to test the autonomous library robot BAR operations, by employing more than 40 books with different page numbers in the lab. Those books were randomly divided into five groups, and each group was placed on shelves of different heights. Both the robot navigation starting points and target books were chosen randomly for testing in the experiments. Once given the commands to access/return any target book, the robot would navigate along the calculated trajectory to the desired place, and then access the book label to perform the BAR operations. The IK model, as illustrated in Equation (17), was adopted to determine the operations of the two robotic arms. Specifically, to test the robot BAR operation accuracies, those parameters obtained from the IK model were also utilized as inputs to the robotic kinematics model, shown by Equation (14). Such results obtained from the robotic kinematics model were finally compared to the coordinates of the book to be accessed for evaluations.

**Figure 19:**
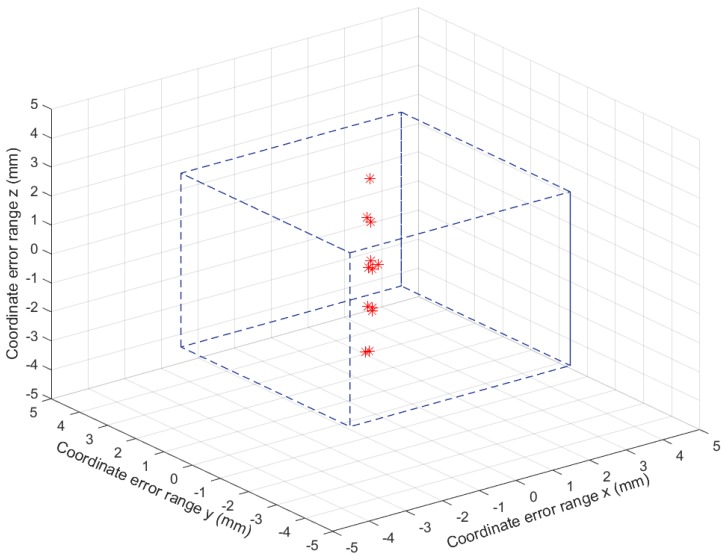
The 20 spatial coordinates fall within the coordinate error range.

Table 6 presents 20 spatial coordinates that were calculated consecutively for a random book by utilizing the IK model solutions as the input of the robotic kinematics model, and Figure 19 shows the calculated coordinate distribution, within the tolerable error range, along three spatial directions. As seen, the coordinates obtained from the robotic kinematic model were very close to the book spatial coordinate (24.0,91.6,276.8), and all the differences between the calculated coordinates and the original book coordinate are within the tolerable error range of ±3 mm. Such results demonstrate that the IK model is effective and robust, and the integration of a binocular vision system and QR code identification technology could help fulfill the desired accuracy of library robot BAR operations. Experiments with returning operations and for books on shelves of different heights also demonstrated the effectiveness of the proposed technology combination in different cases.

## 5. Discussions and Conclusions

In this paper, we report an autonomous mobile robot to fulfill the high-precision BAR operations for modern libraries. The main contributions of this work are two-fold: First, we proposed for the first time (to the best of our knowledge), to integrate binocular vision and QR-code identification technologies together for autonomous mobile robots to improve both P&N and operational accuracies. Second, we designed and constructed a fully autonomous mobile robot for high-precision BAR operations and also verified the applicability of integration of this technology onto the constructed robot. Both simulations and experiments were conducted with the constructed robot to verify the effectiveness of such a technological combination in different cases. Results demonstrated that, with the binocular vision technology adopted for dynamic digital map constructions, autonomous P&Ns, obstacle identification and avoiding, and the QR code identification technique utilized for robot operational error eliminations and robotic arm BAR operation calculations, the robot P&N accuracies could be largely improved and the operation time significantly reduced.

It is worth noting that, within the processes of binocular camera system calibration as well as the object feature extraction and identifications, the binocular vision system imaging background is relatively simple and clean and, therefore, the influences of the environmental factors were omitted in this study. In practice, however, the binocular vision system imaging background could be complicated, and some other practical issues, such as people walking around, the QR-code occlusions, losing or misplacement issues, or too-weak illumination light intensities, would also largely impact on the robot’s performance. Such factors would impose a large burden on the combined binocular vision and QR-code identification mechanism, and sometimes may even cause the QR code label missing issue. Hence, both high-accuracy QR-FEI algorithms and efficient light intensity compensation mechanisms are highly desired to alleviate the influences of such environmental factors. Furthermore, since the main objective of this study was to evaluate the feasibility of the integrated binocular vision and QR-code identification technology for indoor mobile robot BAR operations, only sophisticated existing methods (e.g., the binocular vision based P&N, Haar feature-like extraction based QR-FEI algorithm, and $D^{*}$-Lite algorithm) were deployed. In practice, however, alternative, more efficient methods could also be employed to enable the robot to work more efficiently. The effectiveness and efficiency of such a technological combination also demonstrated its great potential for various other applications. For example, it could be implemented in the companies and laboratories for robots to act as autonomous visitor guides or instructors, or in supermarkets, bookstores, and airports for robots to act as servants or load carriers. Currently, we are evaluating and verifying the applicability of such an integrated binocular vision and QR-code identification technology to an autonomous fruit harvesting robot.

It is also worth noting that, in this study, both the robot hardware fabrication and the software design were implemented according to our lab conditions. The overall cost of such a robot was less than 15K USD, while the average time spent for each BAR operation conducted in the lab was measured to be 2.6 min. In practice, however, if such a robot was batch produced for practical applications, the robot cost could be further reduced, while the average BAR operation time could also be shortened. This is because the robot could be able to manipulate multiple books each time in practice. As compared to a human librarian (typically with a salary of ∼1K USD per month), such a robot could not only help save the labour cost significantly, but also save the time required for BAR operations, especially when the number of books to be processed is large. Long working times and ease of maintenance are the other two advantages of the robot. Due to limited authority to access 3D coordinate information of any public libraries, however, the applicability of such a robot to any real libraries still has to be tested. Currently, we are applying for the permissions to verify the robot applicability within our university libraries.

Our future research work would cover two main aspects. First, sophisticated mechanisms for addressing those issues within real libraries, such as people walking around, the QR-code occlusions, loss or misplacement issues, and too-weak illumination light intensities, have to be developed to improve the robustness of the robot system. Second, more efficient identification and recognition algorithms are to be devised to further improve the P&N and BAR operation accuracies while reducing the system complexity and computational load. In addition, we are also extending the application areas of such an integrated binocular vision and QR code identification technology.

## Figures and Tables

**Table 1:** The modified DH parameters for a single robotic arm.

Link No.	Joint Angle $\theta_{i}$	Link Offset $d_{i}$ (cm)	Link Length $a_{i}$ (cm)	Torsion Angle $\alpha_{i}$
1	$\theta_{1}$	0	0	0
2	$\theta_{2}$	0	0	−90°
3	$\theta_{3}$	0	17.46	0
4	$\theta_{4}$	20	2	−90°

**Table 2:** Binocular vision system parameters.

	Cameras	Left Camera		Right Camera	
Parameters		Central Value	Error Range (±)	Central Value	Error Range (±)
Focal length *f*		[993.26787 992.43228]	[4.01299 4.18855]	[984.20729 977.31964]	[3.33835 3.23414]
Principal point		[330.55970 203.43986]	[4.07621 5.80814]	[299.56229 260.10626]	[5.570527 4.04667]
Skew: $\alpha_{right}$		0.00000	0.00000	0.00000	0.00000
Angles of pixel axes		90.00000	0.00000	90.00000	0.00000
Distortion: $kc_{right}$		[0.03398 0.74095 −0.2645 −0.00608 0.00000]	[0.02857 0.25087 0.00277 0.00212 0.00000]	[0.18789 −0.49039 0.00654 −0.01838 0.00000]	[0.03149 0.23166 0.00187 0.00329 0.00000]

**Table 3:** Binocular vision system calibration results.

	Central Values	Error Range (±)
Rotation vector **R**	[0.05895 0.04133 −0.00169]	[0.00677 0.00690 0.00028]
Translation vector **T**	[62.10517 −0.23686 −2.96750]	[0.24132 0.20697 1.58224]

**Table 4:** X, Y, and Z coordinates of a point in base coordinate system, calculated five times.

	X (mm)	Y (mm)	Z (mm)
1	23.843949	91.087583	273.609231
2	24.013999	91.216968	274.830701
3	24.121204	91.494224	274.830701
4	24.229371	91.626013	276.063126
5	24.507870	91.904512	277.306653

**Table 5:** Binocular vision system versus conventional method.

	Successful Times	Failed Times	Number of Obstacles Hit	Time Used for BAR Operations
Global planning algorithm adopted	8	2	5	321
Binocular vision system	10	0	0	246

**Table 6:** The 20 spatial coordinates determined with the inverse kinematics (IK) model for a book.

x (mm)				y (mm)				z (mm)			
1	23.843949	11	24.618266	1	91.087583	11	92.038743	1	273.609231	11	277.306653
2	24.013999	12	24.618266	2	91.216968	12	92.038743	2	274.830701	12	277.306653
3	24.121204	13	24.618266	3	91.494224	13	92.038743	3	274.830701	13	277.306653
4	24.229371	14	24.618266	4	91.626013	14	92.038743	4	276.063126	14	277.306653
5	24.229371	15	24.72966	5	91.626013	15	92.318496	5	276.063126	15	277.306653
6	24.229371	16	24.72966	6	91.626013	16	92.318496	6	276.063126	16	277.306653
7	24.50787	17	24.72966	7	91.904512	17	92.318496	7	276.063126	17	277.306653
8	24.50787	18	24.898019	8	91.904512	18	92.455208	8	276.063126	18	278.561435
9	24.50787	19	25.010679	9	91.904512	19	92.455208	9	276.063126	19	278.561435
10	24.50787	20	25.010679	10	92.038743	20	92.455208	10	276.063126	20	278.561435

## Abbreviations

The following abbreviations are used in this manuscript:

P&N	Positioning & Navigation
BAR	Book Accessing & Returning
QR	Quick Response
3D	Three-dimensional
QR-FEI	QR code Feature Extraction and Identification
LT	Linear Transformation
PTM	perspective Transformation Matrix
UAV	Unmanned Aerial Vehicle
RFID	Radio Frequency Identification
IK	Inverse Kinematics
